# Feasibility and Safety of Ex Vivo Delivery of Rituximab to Lung Allografts in Transplant Recipients at High Risk for Epstein-Barr Virus–associated Posttransplant Lymphoproliferative Disorder

**DOI:** 10.1097/TXD.0000000000001784

**Published:** 2025-03-28

**Authors:** Victor H. Ferreira, Rafaela V. P. Ribeiro, Faranak Mavandadnejad, Matthew Ierullo, Beata Majchrzak-Kita, Aizhou Wang, Lianne Singer, Shaf Keshavjee, Marcelo Cypel, Deepali Kumar, Atul Humar

**Affiliations:** 1 Ajmera Transplant Centre, University Health Network, Toronto, ON, Canada.; 2 Toronto General Hospital Research Institute (TGHRI), Toronto, ON, Canada.; 3 Department of Laboratory Medicine and Pathobiology, University of Toronto, Toronto, ON, Canada.; 4 Department of Medicine, University of Toronto, Toronto, ON, Canada.; 5 Department of Surgery, University of Toronto, Toronto, ON, Canada.

## Abstract

**Background.:**

Ex vivo lung perfusion (EVLP) offers a novel platform for delivering targeted therapies directly to donor lungs before transplantation, potentially reducing systemic side effects. Our study evaluated the feasibility and safety of rituximab delivery to donor lungs from Epstein-Barr virus (EBV)-seropositive donors for transplantation into EBV-seronegative recipients (D^+^/R^–^) to reduce the risk of EBV-associated posttransplant lymphoproliferative disorder (PTLD), which remains a major obstacle in the transplant setting.

**Methods.:**

A pilot study was conducted involving 5 EBV-seronegative lung transplant recipients. Donor lungs were perfused with 500 mg rituximab during EVLP for 3–4 h. Primary outcomes included safety and feasibility, assessed by monitoring lung function during perfusion, posttransplant complications, and graft dysfunction. Secondary outcomes included EBV DNAemia, PTLD incidence, peripheral B-cell frequencies, EBV blood transcripts, and rituximab serum levels.

**Results.:**

Rituximab delivery via EVLP was feasible and safe, with no significant deviations in lung function or adverse events linked to treatment. One patient experienced primary graft dysfunction. Peripheral B-cell counts were reduced immediately posttransplant and remained low in some patients, whereas others rebounded over the weeks posttransplant, and serum rituximab levels were undetectable after 2 wk. Three patients developed EBV DNAemia and 2 developed PTLD within 2 y, although PTLD lesions were not observed in transplanted lungs.

**Conclusions.:**

EVLP-based rituximab delivery is a feasible and promising strategy for targeting donor-transmitted EBV with minimal systemic exposure. Although the findings suggest potential clinical benefit, the development of PTLD in extrathoracic sites underscores the need for further optimization and larger studies to evaluate efficacy and refine the intervention.

Ex vivo lung perfusion (EVLP) is a novel method of organ preservation that allows donor lungs to be treated under protective physiological conditions before lung transplantation. It has increased access to donor allografts by allowing for wider use of extended criteria lungs.^1^ More recently, it has emerged as a modality that can be used to treat donor organs before transplantation. One such application includes using EVLP to deliver antiviral therapies to organs from seropositive donors before transplantation. Strategies to reduce the viral burden within the allograft before lung transplantation may mitigate downstream deleterious effects. In past studies, our team demonstrated the potential utility of EVLP as an opportunity to deliver monoclonal immunotherapy,^2^ fusion toxin proteins,^3^ and light-based therapy^4^ to donors organs to provide specific antiviral effects against Epstein-Barr virus (EBV), human cytomegalovirus (HCMV), and hepatitis C virus, respectively. Thus far, no clinical study has been conducted to evaluate the efficacy of using EVLP to deliver monoclonal antibody therapy directly to donor organs, particularly to prevent or treat donor-derived viral infections before transplantation.

EBV is a ubiquitous herpesvirus, infecting approximately 90% of the global adult population. After primary exposure, EBV establishes lifelong infection (latency) in B-lymphocytes. Viral reactivation is common after transplantation due to the use of immunosuppression, among other factors, and it can lead to posttransplant lymphoproliferative disorder (PTLD), an aggressive and often lethal type of lymphoma.^5^ Although most organ transplant recipients are EBV seropositive, up to 10% of adult solid organ transplant (SOT) recipients, and a higher percentage of pediatric recipients, are EBV seronegative. In these patients, EBV can be transmitted from a seropositive donor to a seronegative recipient (D^+^/R^−^),^6^ a setting which is associated with a much higher risk of PTLD due to transmission of the virus via the allograft into a naive recipient. In a recent review of a large cohort of EBV D^+^/R^−^ adult SOT recipients from our center, 17.6% developed PTLD, with the highest rates observed for lung transplant recipients (LTRs).^5^ In these cases, the usual pathogenesis of PTLD relates to EBV-driven B-cell proliferation, with the virus being of donor origin.^7^ Because the virus is primarily in its latent form in resident B cells in the graft, conventional antivirals may be of limited benefit, as they would only work to reduce replicative virus, not eliminate the latent virus.

Rituximab is a chimeric monoclonal antibody that targets the CD20 antigen, which decorates the surface of B cells. It is used clinically to treat a variety of pathologies, including hematological cancers and autoimmune disorders. Rituximab has been used successfully as prophylaxis and preemptive therapy for the reduction of EBV DNAemia and PTLD in hematopoietic stem cell transplant (HSCT) recipients.^8-11^ However, systemic administration of rituximab can lead to infusion reactions and increases the risk of infections, and hypogammaglobulinemia and neutropenia have been noted as potential toxicities.^12^ Local, targeted delivery of rituximab to EBV-bearing cells in donor organs could decrease the number of B cells within the graft and mitigate or decrease the effect of donor-transmitted EBV. It would also allow local delivery of a higher dose and obviate the need for systemic administration, potentially improving the safety profile of EBV treatment.

In a prior preclinical study, we found that delivery of rituximab during EVLP led to successful binding of the monoclonal to B cells in lung tissue and in lung lymph nodes, and we observed no apparent safety concerns, suggesting that this could be a new approach for targeting EBV in high-risk transplant recipients. Herein, we describe the results of a pilot safety and feasibility clinical study evaluating this approach in in high-risk EBV D^+^/R^–^ LTRs.

## MATERIALS AND METHODS

### Study Protocol and Sample Collection

**FIGURE 1.**
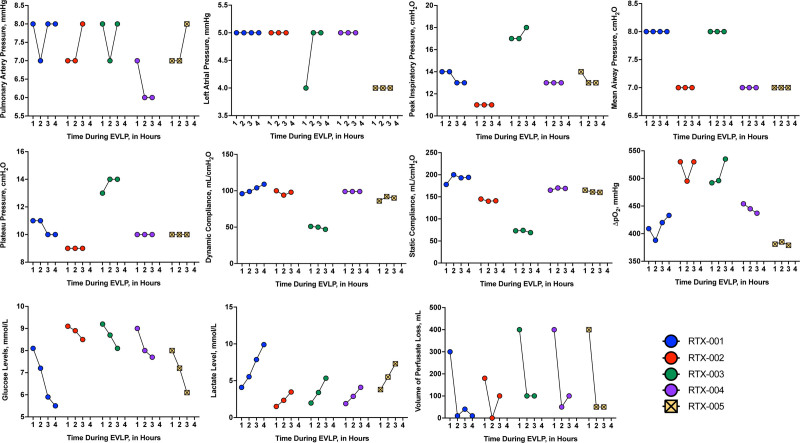
EVLP parameters during rituximab perfusion. During rituximab perfusion, standard EVLP parameters were measured hourly to assess the impact on the lung allograft. Pulmonary artery pressure, left atrial pressure, peak inspiratory pressure, mean airway pressure, dynamic compliance, static compliance, change in partial pressure of oxygen, glucose levels, lactate levels, and volume of perfusate lost in the organ were measured hourly. EVLP, ex vivo lung perfusion.

EBV-seronegative patients were identified from lung transplant candidates listed for transplantation. Patients were approached at a clinic visit to consent for the study. All participants provided written informed consent. The University Health Network (UHN) research ethics board number for the approved study was 19-6260. The study was registered on ClinicalTrials.gov (NCT04507477). Inclusion criteria included age older than 18 y, being listed for single or double lung transplantation, and being EBV seronegative (EBV nuclear antigen IgG and viral capsid antigen IgG; confirmed within the last 30 d). Exclusion criteria included EBV seropositivity at any time before transplant, a history of cancer, history of receiving rituximab or allergy to rituximab, underlying immunodeficiency (eg, common variable immune deficiency), and being unable or unwilling to comply with study procedures. Donor lungs from EBV-seropositive donors deemed potentially suitable for transplant underwent EVLP as per standard protocol. On the ex vivo circuit, donor lungs were treated with 500 mg of rituximab delivered to the circuit perfusate and perfused for 3 or 4 h after an initial hour of EVLP circulation. The rationale for this dose is based on preliminary data demonstrating safety and efficacy.^2^ It is important to note that rituximab was added after the first hour of EVLP (routine assessment window). Additionally, all treated lungs were assessed for their suitability for transplant according to the standard EVLP protocol at our center. Our protocol considers a diverse array of objective and physiological parameters, including those shown in Figure 1. Deteriorations (>15%) in lung physiological parameters, including airway pressures, compliance or pulmonary artery pressure, or a last hour ΔP/F ratio <350 mm Hg, are considered deviations from those normally accepted for transplant.^13^ Lungs were subsequently transplanted using standard procedures. After lung transplantation, patients were followed for 2 y, other than those who died during the study period, and evaluated as per standard of care.

Recipient EBV viral loads were performed weekly posttransplant for the first 4 wk, then monthly for a total of 1 y. Peripheral blood mononuclear cells were collected for B-cell analysis 24 h after transplant (D+1) and at 2 and 4 wk posttransplant. Serum was collected for rituximab measurements at D+1, weekly for 4 wk, and later at month 3. Whole blood was collected in PAXgene tubes to preserve RNA and cryopreserved; samples were thawed before RNA extraction with the RNeasy kit from Qiagen. EBV RNA transcripts were measured at week 2, week 4, and month 3 posttransplant by probe-based real-time reverse transcription polymerase chain reaction (RT-PCR).

The purpose of the study was to collect data on safety, feasibility of recruitment, and implementation of the intervention in the setting of human lung transplantation. The primary outcome was safety, measured as time to extubation and development of primary graft dysfunction (PGD) using previously defined criteria.^14^ Secondary outcomes included development of EBV DNAemia, defined as a plasma EBV viral load ≥1500 IU/mL within the first 12 mo posttransplant; blood B-cell frequencies, measured by flow cytometry; serum rituximab levels, measured by commercial ELISA; and EBV transcript expression in peripheral blood, measured by real-time RT-PCR.

### EBV Clinical Viral Load Testing

Blood was collected and processed for testing at the Mount Sinai-UHN Microbiology Laboratory using the Altona AltoStar EBV-PCR Kit 1.0, with a limit of quantitation of 1500 IU/mL and a limit of detection of 320 IU/mL. Detectable values below the limit of quantitation were set at 750 IU/mL. Undetectable viral loads were plotted at 160 IU/mL.

### Serum Rituximab ELISA

Blood was collected in red-top tubes and allowed to clot for a minimum of 30 min before centrifugation and serum collection. Sera were cryopreserved for batch processing and tested for rituximab spillover into the systemic circulation using a commercial ELISA from MyBioSource. The linear range of detection for the assay was 1.56–100 ng/mL. Values below the limit of quantification were approximated to 0.78 ng/mL.

### Probe-based Real-time RT-PCR for Detecting EBV Transcripts

Whole blood was collected in PAXgene tubes to cryopreserve RNA. After thawing, RNA was extracted and a 1-step, probe-based, real-time RT-PCR assay was performed using the SuperScript III Platinum One-Step RT-PCR Kit (ThermoFisher Scientific). After validation of different primer/probe assays using Raji cells (a kind gift from Dr. Donald Branch lab), the top 3 performing assays were selected, which detected EBV BVRF2 (a late transcript), latent membrane protein 1 (LMP1) and the noncoding RNA, EBER1. The following primers and probes were used: BVRF2 Fwd: CCACGGCAGTCTACGGTACA; BVRF2 Rev: GCGGCATTGGCGTCAT; BVRF2 probe: FAM-ACCTTGCGTGGGTCCTGAAGCACTT-BHQ; LMP1 Fwd: AATTTGCACGGACAGGCATT; LMP1 Rev: AAGGCCAAAAGCTGCCAGAT; LMP 1 probe: FAM-TCCAGATACCTAAGACAAGTAAGCACCCGAAGAT-BHQ; EBER1 Fwd: TGCTAGGGAGGAGACGTGTGT; EBER1 Rev: TGACCGAAGACGGCAGAAAG; and EBER1 probe: FAM-AGACAACCACAGACACCGTCCTCACCA-BHQ. Primer and probe sequences are based on the B95-8 reference genome and were previously published.^15,16^ Detection of EBV transcripts was defined in terms of amplification with a Ct value of ≤38.

### Flow Cytometry

Peripheral blood mononuclear cells were isolated using Ficoll-based separation and cryopreserved. After thawing, cells were stained with Zombie Aqua (BioLegend) viability dye, followed by TruStain Fc receptor blocking agent (BioLegend), and surface antibody incubation with anti-CD20 clone 2H7-BUV395, anti-CD3^−^BV786, and anti-CD19^−^PerCP-Cy5.5. After fixation and permeabilization, cells were incubated with a permeabilization buffer containing CD20 clone 1412-APC. All antibodies were purchased from BD Biosciences. The 2 different CD20 antibodies were selected because clone 2H7 binds surface CD20 epitopes that compete with rituximab, whereas clone 1412 detects a cytoplasmic epitope of CD20, allowing us to explore whether CD20 is being depleted (both clones downregulated) or if it is coated with rituximab or downregulated from the surface (cells will only have clone 1412 staining). Data were acquired on a BD Symphony instrument in the UHN-Sickkids Flow Cytometry Facility. Fluorescence-minus one controls were used to guide gate setting. A minimum of 10 000 live CD3^−^ cells were analyzed in each sample to provide robust data.

## RESULTS

### Patient Enrollment and Overall Outcomes

A total of 5 EBV D^+^/R^–^ patients were enrolled. Individual patient characteristics and outcomes are described in more detail below. Four patients underwent lung transplants for chronic obstructive pulmonary disease (COPD) and 1 patient for cystic fibrosis. The median age of transplant recipients was 63.0 y (range, 30–67). The median age of donors was 37.0 y (range, 30–63). All participants received triple immunosuppression. Overall, the logistics of EVLP proceeded smoothly. Airway parameters, namely pulmonary artery pressure, left atrial pressure, peak inspiratory pressure, mean airway pressure, plateau pressure, dynamic compliance, static compliance, and change in partial pressure of oxygen (ΔpO_2_) were measured hourly for each patient, along with glucose and lactate levels, and volume of perfusate lost (Figure 1). All parameters remained stable during rituximab perfusion with no significant deviation from the norm. Posttransplant, no attributable adverse events were noted. Rituximab in serum was detected in at least 1 time point in 4 of 5 participants. EBV DNAemia was detected in 3 of 5 patients. PTLD occurred in 2 of 3 patients with documented EBV DNAemia. No antivirals were used specifically for EBV DNAemia, and immunosuppression was not modified in these patients on the basis of EBV DNAemia.

### Participant RTX-001

Patient RTX-001 was a 65-y-old man who underwent double lung transplantation for the underlying history of COPD. Their donor was a 37-y-old man who experienced neurological determination of death related to trauma. Posttransplant, RTX-001 was extubated within 48 h. Postoperative complications included atrial fibrillation and possible pneumonia due to methicillin-sensitive *Staphylococcus aureus* and Enterobacter, for which the patient was treated with antibiotics (cefazolin and ciprofloxacin). No PGD was documented after transplantation. The intensive care unit (ICU) length of stay was 2 d and hospitalization was 25 d. Immunosuppression for RTX-001 included tacrolimus, prednisone, and mycophenolate.

**FIGURE 2.**
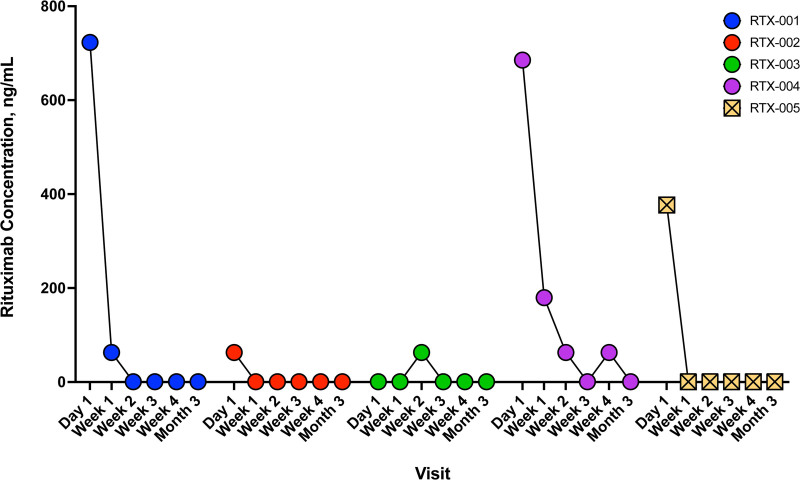
Serum rituximab levels posttransplant. Rituximab levels in sera were measured using a commercial rituximab ELISA and reported in nanogram per milliliter. Each dot represents a sample test result. The dashed horizontal line represents the limit of quantification of the assay.

**FIGURE 3.**
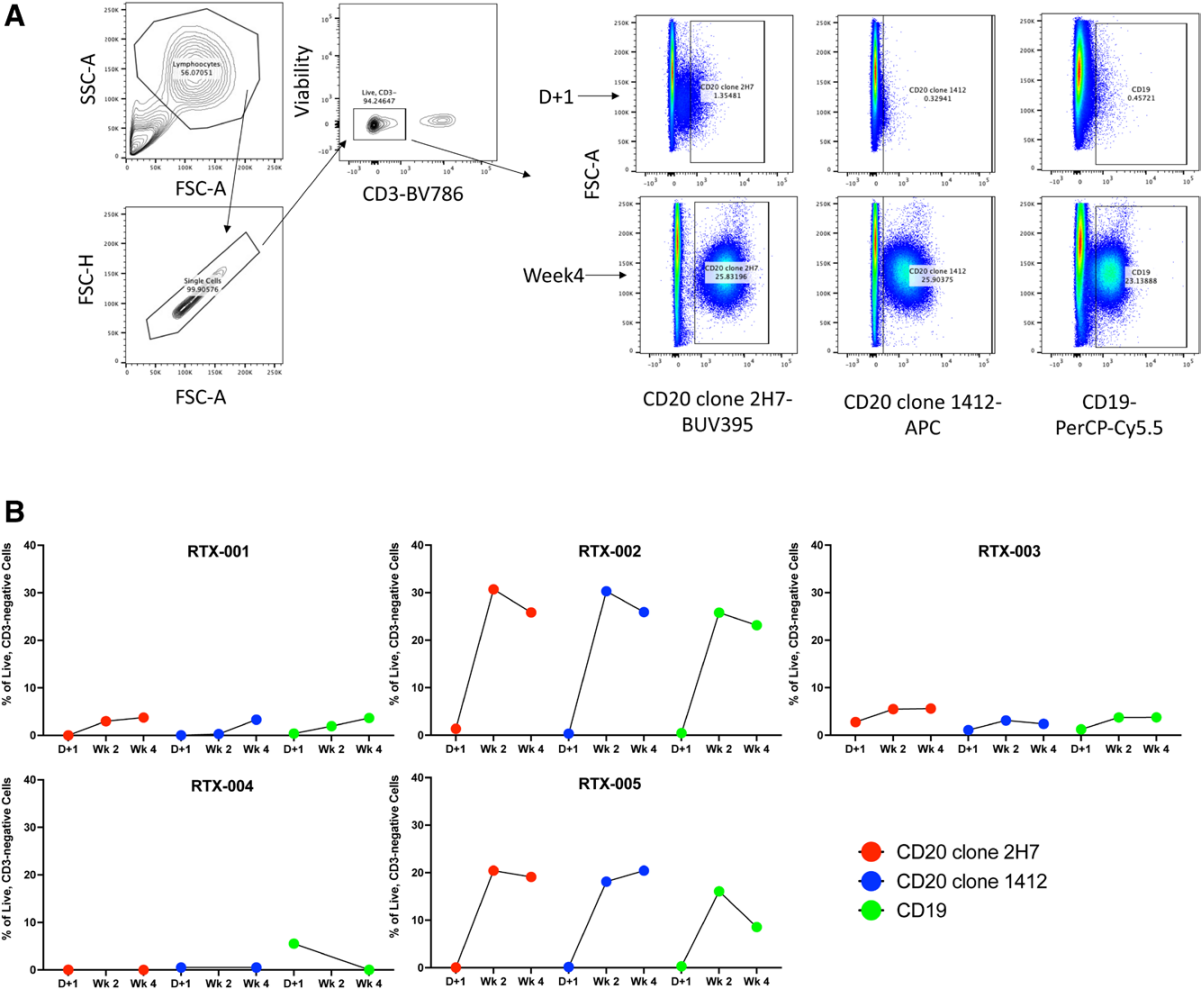
B-cell analysis in peripheral blood post-lung transplantation. A, A representative gating strategy for identifying B cells using flow cytometry. B, B-cell frequencies measured the day after transplantation (D+1) and weeks 2 and 4 posttransplantation. Results are reported in terms of live, CD3^−^ cells. FSC-A/H, forward scatter-area/height; SSC-A, side scatter-area.

Serum rituximab levels were measured to assess potential spillover into the blood (Figure 2). At day 1 posttransplant, serum rituximab levels reached 723 ng/mL and declined to 63 ng/mL by week 1. As of week 2, levels were no longer detectable. Peripheral CD20^+^ or CD19^+^ B cells were largely undetectable on day 1 (Figure 3). Although frequencies increased by week 4, B-cell numbers remained generally low (3%–4% of live, CD3^−^ cells).

**FIGURE 4.**
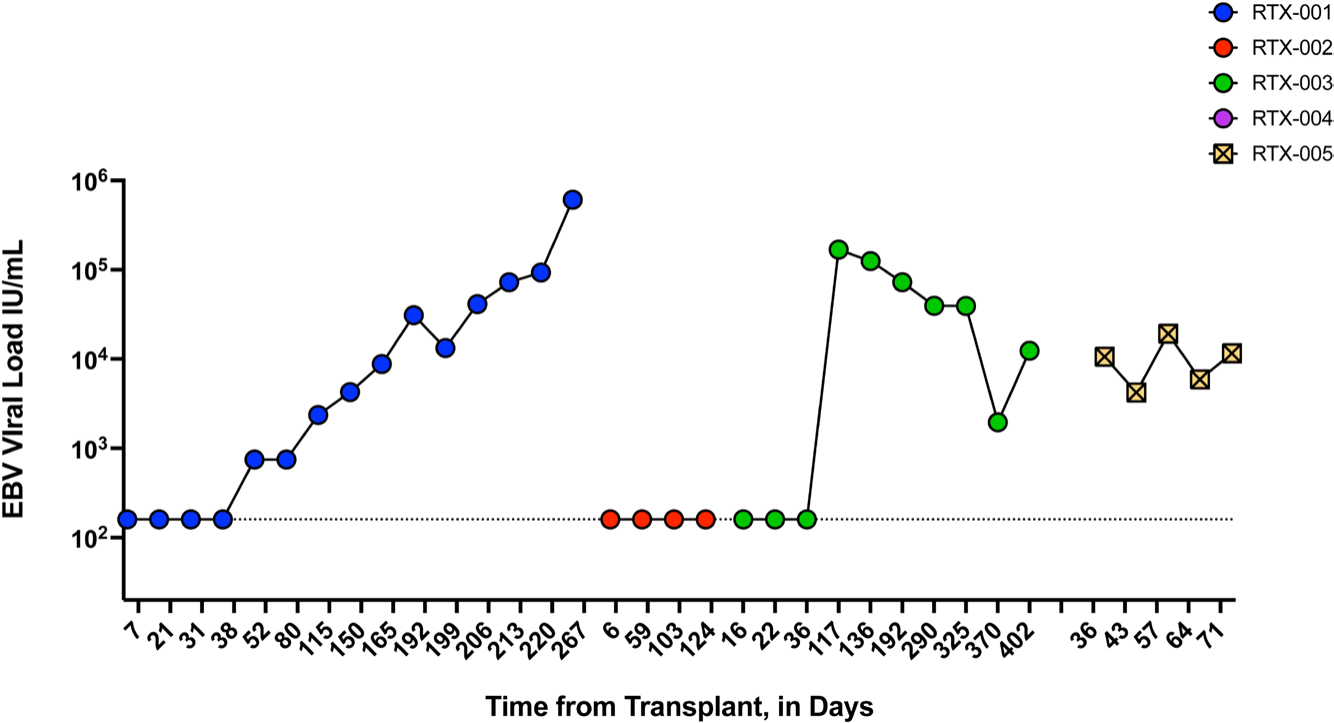
EBV viral loads throughout the study. Clinical viral load measurements are shown, with each circle representing a test result and plotted in terms of international units per milliliter. Figure is color-coded by patient ID. Horizontal dashed line indicates the limit of quantification of the clinical qPCR assay. EBV, Epstein-Barr virus.

**FIGURE 5.**
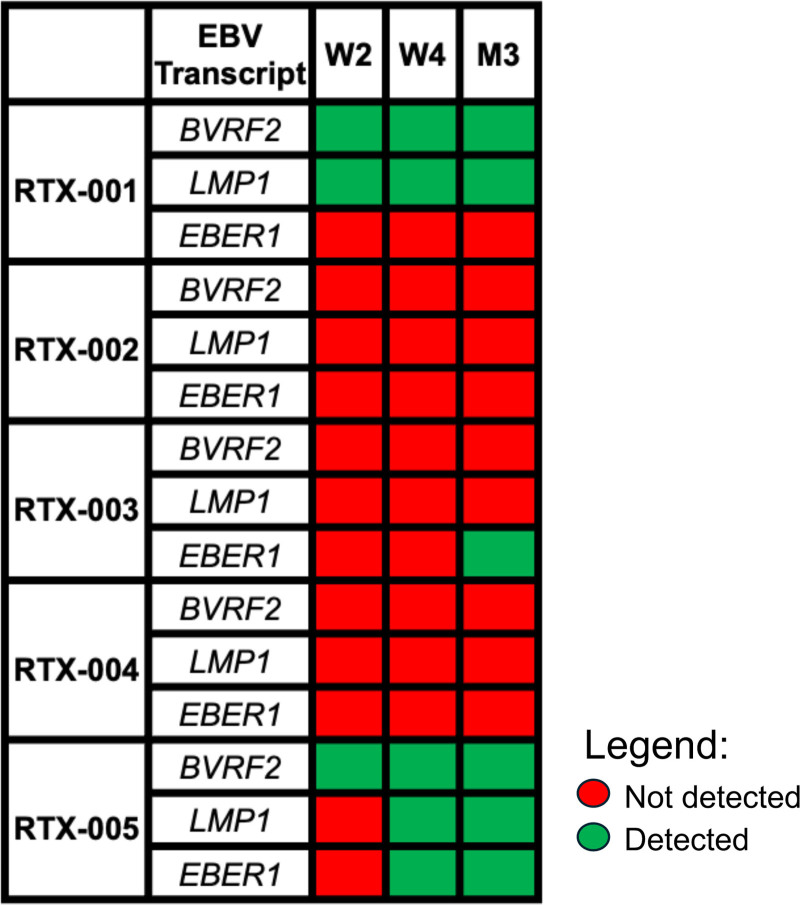
EBV transcript detection post–lung transplantation. Whole blood was collected at week 2 (W2), week 4 (W4), and month 3 (M3) posttransplant and cryopreserved in PAXgene tubes. After thawing, RNA was isolated and probe-based real-time RT-PCR was performed to detect EBV transcripts. Figure is color-coded based on transcript expression at each time point. EBER1, Epstein-Barr virus–encoded small RNA 1; EBV, Epstein-Barr virus; LMP1, latent membrane protein 1.

EBV DNAemia was detected on day 115 posttransplant (Figure 4). Viral loads climbed and stayed persistently detected during follow-up. EBV transcripts (BVRF2 and LMP1) were detected in whole blood at all 3 study time points (Figure 5). PTLD, specifically, monomorphic, EBV-positive diffuse large B-cell lymphoma, was identified on day 255 with lesions detected in the sigmoid colon and liver but none in the lung allograft. Their follow-up course was further complicated by an acute bowel perforation requiring resection, acute kidney injury, and sepsis. The participant died on day 278 posttransplantation with sepsis due to bowel perforation as the cause of death.

### Participant RTX-002

RTX-002 was a 30-y-old woman who underwent double lung transplantation for cystic fibrosis. Her donor was a 37-y-old woman experiencing donation after circulatory death (DCD) related to medical assistance in dying. RTX-002 was extubated within 24 h, and the length of ICU stay and hospitalization were 2 and 19 d, respectively. No PGD was documented. Her posttransplant course was complicated by Scedosporium fungal infection of the pleura and pericardial space, well controlled by antifungals, and HCMV DNAemia, treated with valganciclovir. Immunosuppression consisted of treatment with cyclosporine, prednisone, and mycophenolate.

Rituximab levels in serum were very low on the day after transplantation and undetectable by week 1 (Figure 2). B cells were not detected on the day after transplantation, but frequencies rebounded by week 2, expanding to 25%–30% of live, CD3^−^ cells (Figure 3). EBV was not detected as part of clinical viral load monitoring (Figure 4), and no EBV transcripts were detected in peripheral blood (Figure 5). At a 2-y follow-up, she remained free of EBV DNAemia and PTLD.

### Participant RTX-003

Patient RTX-003 was a 63-y-old woman who underwent double lung transplantation for COPD. Her donor was a 63-y-old woman experiencing DCD due to cardiomyopathy. The transplant recipient was sensitized and had a positive virtual crossmatch. They received induction therapy with thymoglobulin, plasmapheresis, and IVIG; no other participants in the study received induction therapy. Her immunosuppression regimen consisted of cyclosporine, prednisone, and mycophenolate. After the transplant, RTX-003 had prolonged mechanical ventilation due to the development of PGD category 3 and was treated for 4 d on extracorporeal membrane oxygenation. Participants also developed venous thromboembolism, atrial fibrillation, and fungal mediastinitis with pericardial effusion, treated with antifungals. ICU length of stay was 18 d, and hospitalization was 69 d. Around 4 mo posttransplant, HCMV DNAemia was identified, and the participant was treated with therapeutic valganciclovir. On day 117, EBV DNAemia was identified, and viral loads remained elevated throughout the follow-up period (Figure 4). EBER1 transcripts were detected at 3 mo (Figure 5). During the first year, no episodes of PTLD occurred. However, in the second year posttransplant (day 497), EBV^+^ diffuse large B-cell lymphoma PTLD was detected in breast tissue and an axillary lymph node, but the lung remained unaffected. The participant subsequently passed away on day 684 from sepsis relating to a wound infection. Serum rituximab levels were generally not detected in the posttransplant phase; however, a low level was observed in the week 2 sample (Figure 2). B cells were detected at very low levels on day 1 and increased by week 4 but remained generally low in frequency (~5% of live, CD3^−^ cells; Figure 3).

### Participant RTX-004

Participant RTX-004 was a 67-y-old man undergoing double lung transplantation due to COPD. Their donor was a 56-y-old male DCD donor who died from a stroke. RTX-004’s bronchoalveolar lavage culture after transplant showed Aspergillus and Fusarium spp colonization and received voriconazole as a preemptive treatment for 12 wk. Their time to extubation was 48 h, and ICU length of stay was 4 d. A week posttransplant, he developed acute onset hypoxemic respiratory failure and delirium, worsening bilateral infiltrates, grade 1 bilateral pneumothorax, and subcutaneous emphysema. The participant was intubated and extubated the next day and improved. He was discharged from the hospital on day 17. Five months after transplantation, he was admitted for COVID-19, which led to respiratory failure and death. COVID-19 was listed as the cause of death. Clinical EBV PCRs were not available for this participant. However, PAXgene tubes of whole blood were collected, and no EBV transcripts were detected at any time point (Figure 5). Serum rituximab levels were detectable on day 1 posttransplant and remained quantifiable at weeks 1 and 3 (Figure 2). Peripheral blood B cells were minimally detected in this participant on day 1 and week 4 (no week 2 sample was provided; Figure 3).

### Participant RTX-005

Participant RTX-005 was a 62-y-old man who underwent a double lung transplant for COPD. His donor was a 30-y-old man who experienced neurological determination of death related to a drug overdose. RTX-005 had an uncomplicated postoperative course with an ICU length of stay of 4 d and a total hospitalization of 11 d. He developed EBV DNAemia approximately 1 mo after transplant (Figure 4), but no evidence of PTLD occurred during the study period. BVFR2 transcripts were found at 2 wk posttransplant, and all 3 transcripts were measured at week 4 and month 3 in peripheral blood (Figure 5). Serum rituximab was detected at day 1 (377 ng/mL) but declined to unquantifiable levels thereafter (Figure 2). B-cell levels were not detectable on day 1 but rose in the following weeks, with CD20^+^ B-cell frequencies reaching ~20% of live, CD3^−^ cells by week 2 (Figure 3).

## DISCUSSION

There are limited clinical data on the utilization of therapeutic modalities in an ex vivo setting to modify organs. This represents a potentially attractive approach as it allows delivery of active agents at local high doses, with minimal chance of systemic toxicity. Overall, we found that EVLP-based delivery of rituximab to treat EBV was feasible and overall safe. Despite rituximab therapy, EBV DNAemia occurred in 3 of 5 participants, one of who developed PTLD in the first year and a second who developed PTLD in the second year after lung transplantation. However, an interesting observation was that in both patients with PTLD, no lesions were found in the lung allografts, suggesting the ex vivo delivered rituximab may have had a local effect. Previous studies of PTLD in D^+/^R^–^ LTRs have noted that allograft involvement is quite common.^5^

Data evaluating the use of anti-CD20 monoclonal antibodies to prevent EBV DNAemia or PTLD are lacking in SOT patients. Indirect evidence comes from a study of SOT recipients receiving rituximab at induction for ABO incompatibility who were found to develop PTLD at lower rates.^17^ In the HSCT setting, the use of rituximab to prevent PTLD has become an initial preemptive intervention for controlling EBV DNAemia.^18^ Moreover, rituximab used as prophylaxis was associated with a reduction in EBV DNAemia and PTLD in high-risk HSCT recipients.^19^ Single-center experiences have reported a reduction in PTLD rates with rituximab use in SOT recipients compared with historical or contemporaneous controls.^20,21^ More recently, a randomized controlled trial showed that pediatric LTRs who received rituximab had less EBV viral load detection than the placebo group. However, the study was underpowered to document an effect on PTLD rates.^22^

Our results are encouraging but suggest that further modifications to our strategy may be required to prevent PTLD in high-risk LTRs. Prolonged duration of perfusion may facilitate improved attachment and rituximab activity. No PTLD was found in the lung allograft, suggesting that it may have been protected. This observation must be tempered, although, by the smaller sample size in our study and the fact that patients still developed lethal PTLD. Early EBV-positive extrathoracic PTLD may still result from donor-transmitted EBV, and the frequency of PTLD in this small cohort was not necessarily lower than expected. One or more subsequent doses of rituximab, delivered systemically, may provide protection beyond the allograft and bolster the performance of this approach. Other CD20-targeting monoclonals are also available and may provide improved activity compared with rituximab. Additionally, future studies should be performed in younger LTRs, including pediatric recipients, where EBV D^+^/R^–^ transplantations are more common, as is the rate of PTLD. The lack of EBV DNAemia and PTLD in our youngest study participant provides some rationale for this.

Our study had limitations. First, the sample size was small. However, this was primarily a feasibility and safety study to assess a clinical ex vivo therapeutic intervention. Also, the number of adult EBV D^+^/R^–^ lung transplantations performed at our center is low due to high seroprevalence rates of EBV. The cohort represents a significant proportion of eligible participants, particularly as the study was conducted during the COVID-19 pandemic when limitations were placed on transplant services. Larger future studies will be required to evaluate efficacy. We also were unable to collect a pre or peritransplant blood specimen to determine whether decreased B-cell numbers were due to rituximab alone and/or immunosuppression. We did not include a control group; however, historical EVLP data were used to identify whether rituximab treatment caused deviations from expected lung physiological parameters (data not shown). Controls would also help in interpreting the posttransplant EBV and immune parameters, but this was not the primary focus of our pilot study, and in the future, larger trials with refined methodologies are needed to validate virologic outcomes and extend our findings. Additionally, although the strategy of transplanting EBV^–^ lungs aims to reduce the risk of PTLD in EBV^–^ recipients, this approach may not address all sources of primary EBV infection or mitigate the risk of EBV^–^ PTLD.

In summary, we demonstrate a first-in-human study using ex vivo organ perfusion as a platform to deliver monoclonal antibody treatment directly to donor lungs before transplantation. This approach shows potential for reducing the incidence of EBV-related complications, such as PTLD, in high-risk LTRs and also suggests an opportunity to intervene with other immunotherapies during EVLP, providing a unique opportunity to treat donor organs before transplantation. This will lead to improved outcomes for transplant recipients and will extend the number of organs available for waitlisted patients.

## ACKNOWLEDGMENTS

The authors thank Ms Ilona Bahinskaya and Natalia Pinzon (UHN) for clinical study coordination.
